# Biased Diversity Metrics Revealed by Bacterial 16S Pyrotags Derived from Different Primer Sets

**DOI:** 10.1371/journal.pone.0053649

**Published:** 2013-01-14

**Authors:** Lin Cai, Lin Ye, Amy Hin Yan Tong, Si Lok, Tong Zhang

**Affiliations:** 1 Environmental Biotechnology Laboratory, Department of Civil Engineering, The University of Hong Kong, Hong Kong SAR, China; 2 Li Ka Shing Institute of Health Sciences, The Chinese University of Hong Kong, Hong Kong SAR, China; Wageningen University, The Netherlands

## Abstract

In recent years, PCR-based pyrosequencing of 16S rRNA genes has continuously increased our understanding of complex microbial communities in various environments of the Earth. However, there is always concern on the potential biases of diversity determination using different 16S rRNA gene primer sets and covered regions. Here, we first report how bacterial 16S rRNA gene pyrotags derived from a series of different primer sets resulted in the biased diversity metrics. In total, 14 types of pyrotags were obtained from two-end pyrosequencing of 7 amplicon pools generated by 7 primer sets paired by 1 of 4 forward primers (V1F, V3F, V5F, and V7F) and 1 of 4 reverse primers (V2R, V4R, V6R, and V9R), respectively. The results revealed that: i) the activated sludge exhibited a large bacterial diversity that represented a broad range of bacterial populations and served as a good sample in this methodology research; ii) diversity metrics highly depended on the selected primer sets and covered regions; iii) paired pyrotags obtained from two-end pyrosequencing of each short amplicon displayed different diversity metrics; iv) relative abundance analysis indicated the sequencing depth affected the determination of rare bacteria but not abundant bacteria; v) the primer set of V1F and V2R significantly underestimated the diversity of activated sludge; and vi) the primer set of V3F and V4R was highly recommended for future studies due to its advantages over other primer sets. All of these findings highlight the significance of this methodology research and offer a valuable reference for peer researchers working on microbial diversity determination.

## Introduction

Bacteria are capable of populating nearly any living environment on the Earth (e.g. soil, water, air, human body, and even extreme environments) and contribute greatly to the global matter cycle and energy metabolism. Our understanding of bacteria and their roles is largely limited because more than 99% bacterial species cannot be isolated and cultivated with current laboratory practices [1]. Over the past decades, both traditional culture dependent and independent approaches (including isolation, cloning, functional identification, DGGE (denaturing gradient gel electrophoresis) [2], T-RFLP (terminal restriction fragment length polymorphism) [3], FISH (fluorescence *in situ* hybridization) [4], and Genechips [5] etc.) always cause bottlenecks in exploring global bacterial diversities and their potential functions. To overcome these limitations, high-throughput sequencing technologies have been well developed to promote the relevant research fields [6]. The Roche 454, SOLID, Ion Torrent, and Illumina platforms have dominated the next-generation sequencing market and made notable contributions to the genomic and metagenomic studies in the past five years [7]. Roche 454 pyrosequencing technology, a high-throughput platform generating relatively long length reads, has greatly promoted the exploration of bacterial diversities in various environments [6]. Bacterial 16S rRNA gene encodes the small subunit ribosomal RNA molecule that has been thought to be the most versatile and reliable marker gene for profiling bacterial populations. The 16S rRNA gene full length usually contains nine hypervariable regions (V1–V9) flanked by nine highly conserved regions (C1–C9) [8]. PCR-based pyrosequencing of 16S rRNA genes (commonly defined as ‘16S pyrotags’) allows researchers to profile highly complex bacterial community compositions in depth, especially for exploring currently uncultivated and unknown bacteria.

All commonly used 16S rRNA gene universal primers are designed in the highly conserved regions that are believed to exhibit different coverage for different bacterial lineages. This has been revealed by computational simulation (*in silico* prediction) using the collected 16S rRNA gene databases [9]–[11]. Several case studies have also pointed out that primer selections and targeted regions could have a significant impact on the determination of bacterial diversity based on 16S pyrotags from several environmental samples [12]–[14]. However, the biased extent and depth have not been investigated comprehensively. Moreover, the surveyed samples including human feces [12], termite hindgut [13], and smoker subgingival plaque [14] only contained low bacterial diversities and could not represent the highly diverse bacterial populations across a broad range of different environments. In this study, we employed composite activated sludge samples from 11 municipal wastewater treatment plants as the target system to figure out this commonly concerned problem. The activated sludge is a highly complex open system that harbors a variety of bacteria, archaea, fungi, algae, protozoa, and viruses. In this open system, bacteria are absolutely dominant and responsible for the removal of various nutrients and pollutants during wastewater treatment [15]. Hence, colonized bacterial species in activated sludge could provide a widespread bacterial coverage and serve as a representative sample for the comprehensive evaluation in this study.

Employing high-throughput sequencing technology is the current and future trend to explore highly complex bacterial communities and their potential functions in any interested environments. The objective of this study is to investigate how the 16S rRNA gene primer sets and covered regions affect the bacterial diversity metrics. The findings offer a valuable reference on this commonly concerned issue for peer researchers working on bacterial diversity determination using 16S pyrotags, and provide useful information for improving the current 16S rRNA gene pyrosequencing practices.

## Materials and Methods

### Experimental Design

**Figure 1:**
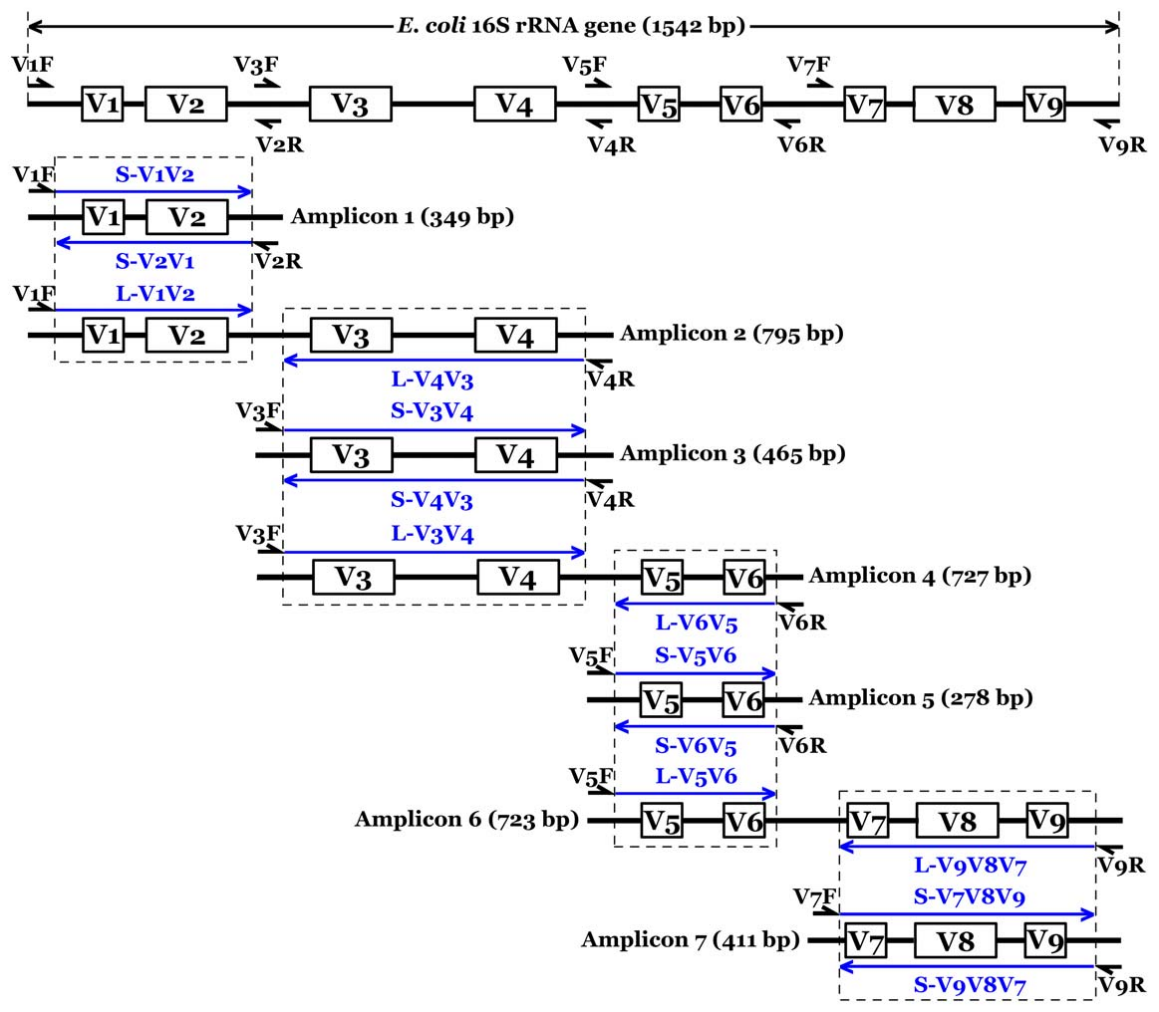
Schematic representation of experimental design employed in this study. The full length of *E. coli* 16S rRNA gene was used as the reference scale. Fourteen blue arrow lines indicated all pyrotags analyzed in this study. S (L)-V1V2 represented the pyrotag contained 16S V1V2 region derived from a Short (Long) amplicon. Four small PCR fragments covered 16S V1V2, V3V4, V5V6, and V7V8V9 regions were defined as Short amplicons. The remainder three large PCR fragments targeted 16S regions of V1–V4, V3–V6, and V5–V9 were defined as Long amplicons.

PCR-based pyrosequencing of 16S rRNA genes was employed to investigate the potential biased diversity metrics. The detailed experimental design of this study is described in Figure 1. To evaluate 16S primer sets and covered regions in regard to bacterial diversity coverage and/or accurate taxonomic assignment, 4 forward primers (V1F, V3F, V5F, and V7F) and 4 reverse primers (V2R, V4R, V6R, and V9R) targeting 16S rRNA gene different regions were paired to generate 4 short amplicons covering V1V2, V3V4, V5V6, and V7V8V9, and 3 long amplicons covering V1–V4, V3–V6, and V5–V9. After individual PCR amplification, a total of 7 fragments (named as Amplicon 1–7) ranging from 278 to 795 bp in length were obtained, as shown in Table 1 and Figure 1. To minimize the unspecific amplification and investigate the potential biases of 454 pyrosequencing technology, each 16S primer only carried the oligonucleotide barcodes at the 5′ terminus, but without 454 adaptors. After PCR, the purified products were ligated with 454 A- and B-adaptors using TA ligation strategy. Thus, the priming 454 A-adaptor was randomly modified to two ends of the PCR fragments, and each amplicon could be pyrosequenced twice (i.e. one pyrotag was sequenced from the end of 16S forward primer and the other pyrotag was sequenced from the end of 16S reverse primer). Finally, the 7 amplicons generated 14 pyrotags for subsequent bioinformatic analysis. They were designed as eight S-type pyrotags (S-V1V2, S-V2V1, S-V3V4, S-V4V3, S-V5V6, S-V6V5, S-V7V8V9, and S-V9V8V7) and six L-type pyrotags (L-V1V2, L-V4V3, L-V3V4, L-V6V5, L-V5V6, and L-V9V8V7). S-type and L-type pyrotags represented pyrotags derived from 4 short amplicons and 3 long amplicons, respectively.

**Table 1:** Primers used in this study.

Names	Barcodes(5′-3′)	Sequences(5′-3′)	Positions	References
V1F	***G*** AGCACTGTAG	AGAGTTTGATCCTGGCTCAG	8–27	[36], [37]
	***G*** TAGTATCAGC			
V3F	***G*** ACTACTATGT	ACTCCTACGGGAGGCAGCAG	338–357	[32], [33]
	***G*** TGATACGTCT			
V5F	***G*** TCGTCGCTCG	ATTAGATACCCNGGTAG	787–803	[38], [39]
	***G*** ATATCGCGAG			
V7F	***G*** CGTGTCTCTA	GYAACGAGCGCAACCC	1099–1114	[13], [40]
V2R	***G*** ATCAGACACG	TGCTGCCTCCCGTAGGAGT	338–356	[36], [37]
V4R	***G*** CTCGCGTGTC	TACNVGGGTATCTAATCC	785–802	[35], [41]
	***G*** ACGAGTGCGT			
V6R	***G*** AGACTATACT	CGACAGCCATGCANCACCT	1046–1064	[42], [43]
	***G*** ACGCTCGACA			
V9R	***G*** CATAGTAGTG	GNTACCTTGTTACGACTT	1492–1509	[38], [39]
	***G*** TACTGAGCTA			

The positions of all primers are referred to the 16S rRNA gene of *E. coli* str. K12 substr. DH10B. The bold italic nucleotide ‘*G*’ fused into the 5′ terminus of each barcode is to offer an unbiased ligation between amplicons and 454 adaptors.

### Sampling and DNA Isolation

In this study, activated sludge samples were collected from 11 municipal wastewater treatment plants: Singapore (1), Hong Kong (2), North America (3), and Mainland China (5). These samples were taken from their respective aeration tank and immediately mixed with 100% ethanol at a 1∶1 volume ratio to fix microbial cells. Samples were kept and transported using ice bath before reaching this laboratory and stored at −20°C. The detailed characteristics of these wastewater treatment plants and samples were illustrated in a previous publication [16]. No specific permits were required for the described field studies. We confirm that: i) the locations were not privately-owned or protected in any way; and ii) the field studies did not involve endangered or protected species.

The FastDNA® SPIN Kit for Soil (MP Biomedicals, France) was used to extract total DNA from the collected samples individually according to the manufacturer’s manual. This kit was the most suitable to isolate total DNA from activated sludge compared with other commercial kits by empirically judging from the purity and PCR ability of the DNA extracts. The concentration of each DNA extract was determined by Thermo NanoDrop 1000 Spectrophotometer. The 11 DNA extracts were pooled with equal mass to obtain one mixed DNA sample used as the PCR template.

### PCR and 454 Pyrosequencing

The PCR amplification was performed by BioRad i-Cycler under conditions of initial 5 min denaturation at 94°C; 35 cycles of 50 sec at 94°C, 30 sec at 40°C, and 90 sec at 72°C; and final 5 min extension at 72°C. Each standard PCR volume contained 50 ng DNA, 200 nM of each primer (Integrated DNA Technologies, US), 200 µM dNTPs (Invitrogen, US), 2 mM MgSO_4_, 5 µL of 10 × High Fidelity PCR Buffer, 0.2 µL Platinum *Taq* High Fidelity (Invitrogen, US), and ddH_2_O up to 50 µL. Triplicate amplification was conducted for each amplicon. The products from the same amplicons were pooled together (∼150 µL) for subsequent purification using PCRquick-spin™ PCR Product Purification Kit (iNtRON Biotechnology, Korea). The purified amplicons were quantified by Thermo NanoDrop 1000 Spectrophotometer and mixed at a mole ratio of 1∶1.5 for short amplicons *vs* long amplicons. The pooled sample was submitted to the Genome Research Centre at The University of Hong Kong (GRC at HKU) to complete 454 adaptors ligation and pyrosequencing on the Roche 454 FLX Titanium platform (Roche, US).

### Sequence Trimming and Bioinformatic Analysis

All pyrosequencing reads were strictly trimmed and quality-controlled. The 454 adaptors at the terminus of raw reads were firstly removed by the staff of GRC at HKU. Then, 14 types of pyrotags were assigned into different files based on the exact matching with the unique barcodes. The Ribosomal Database Project (RDP) Pyrosequencing Pipeline Initial Process Tool [17] was employed to trim reads as follows: i) any ambiguous base was not allowed, ii) reads shorter than 200 bp were filtered off, and iii) reads containing both forward and reverse primers were kept after the primer sequences were checked and removed. Afterwards, the Chimera Slayer [18], [19] was used to remove potential chimeras, and pre.cluster [20], [21] was further applied to denoise. Finally, the clean pyrotags obtained after filtration were used for the downstream bioinformatic analysis.

In this study, both OUT-based (operational taxonomic unit) and classification-based analyses were performed by RDP online tools. After sequence trimming processed, jobs of alignment, complete linkage clustering, rarefaction, Shannon diversity, and Chao1 richness were all finished by RDP’s Pyrosequencing Pipeline. RDP Classifier, based on Naive Bayesian algorithm [22], was carried out to assign all clean pyrotags into each taxonomic unit (from phylum to genus) at a confidence threshold of 50% cutoff. The generated taxonomic data were summarized into an Excel table and visualized using OriginPro 8 software. The PAST statistical software package [23] was used to perform cluster analysis for all pyrotags.

## Results

### Overview of Processed Pyrotags and Overall Bacterial Diversity

A total of 939,075 raw reads were produced in a complete run by Roche 454 pyrosequencing platform. After following strict criteria by filtering the reads of low quality, noise, and chimera, the number of accepted reads decreased dramatically with only 395,718 reads passed for the downstream analysis. The detailed filtering data, 97% OTU numbers, Shannon and Chao1 indices for each pyrotag were summarized in Table 2. Considering the highly different numbers of clean reads between S-type and L-type pyrotags, the two types of pyrotags were compared at different sequencing depths (23,609 for S-type and 3,276 for L-type) by subsampling the first 23,609 reads and 3,276 reads for S-type and L-type pyrotags, respectively. Analyzing all available clean pyrotags was also performed and attached in the Supporting Information.

**Table 2:** Overview of processed pyrotags.

Pyrotags	Number of raw pyrotags	Number of clean pyrotags	Average length (bp)	97% cutoff OTUs	Chao1 richness	Shannon diversity
S-V1V2	59940	36262	302	8223	19423	7.40
S-V2V1	69008	37293	303	7975	17522	7.37
S-V3V4	60061	32339	415	12326	40858	8.30
S-V4V3	66651	37853	415	13497	41773	8.26
S-V5V6	117035	78807	242	20456	66513	8.01
S-V6V5	162100	69337	242	20468	65285	8.17
S-V7V8V9	67198	42996	373	16238	52871	8.35
S-V9V8V7	38669	23609	373	9676	33611	8.03
L-V1V2	29916	4646	299	1786	4009	6.70
L-V4V3	26487	4468	413	2317	8977	7.14
L-V3V4	31663	3276	415	1658	6072	6.81
L-V6V5	35945	7311	242	2942	10901	7.02
L-V5V6	15539	8621	242	4085	15631	7.51
L-V9V8V7	19940	8900	373	3735	12120	7.34
No barcode	138923	N/A	N/A	N/A	N/A	N/A
Total	939075	395718	N/A	N/A	N/A	N/A

The raw pyrotags were the original reads generated by the 454 run. After removal of low quality reads, short reads, chimera, noise, and archaea reads, the clean pyrotags were finally obtained and used for downstream bioinformatic analysis.

**Figure 2:**
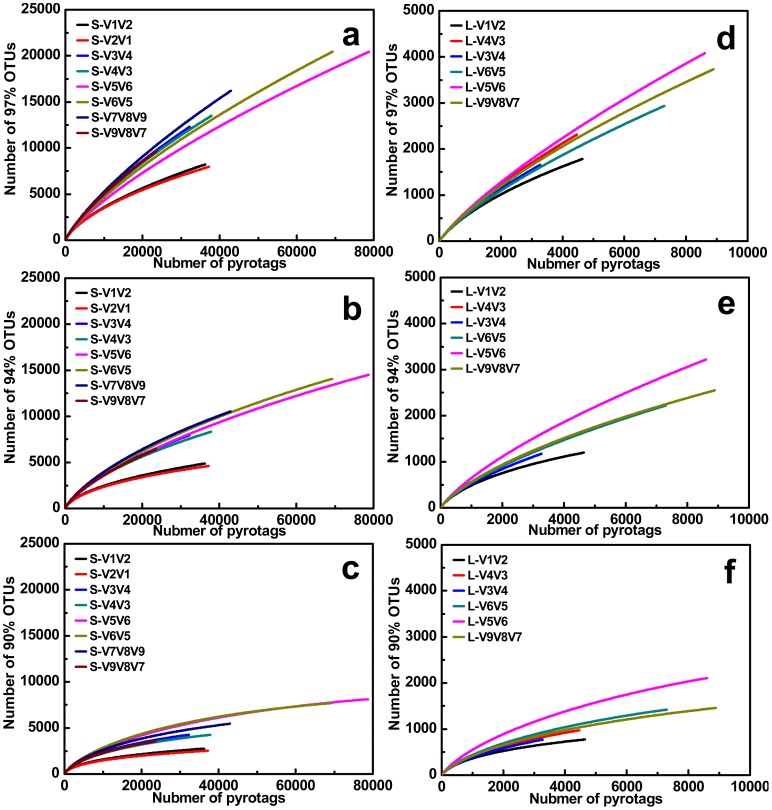
Rarefaction curves of 97%, 94%, and 90% OTUs for eight S-type pyrotags (S-V1V2, S-V2V1, S-V3V4, S-V4V3, S-V5V6, S-V6V5, S-V7V8V9, and S-V9V8V7) and six L-type pyrotags (L-V1V2, L-V4V3, L-V3V4, L-V6V5, L-V5V6, and L-V9V8V7), respectively.

**Figure 3:**
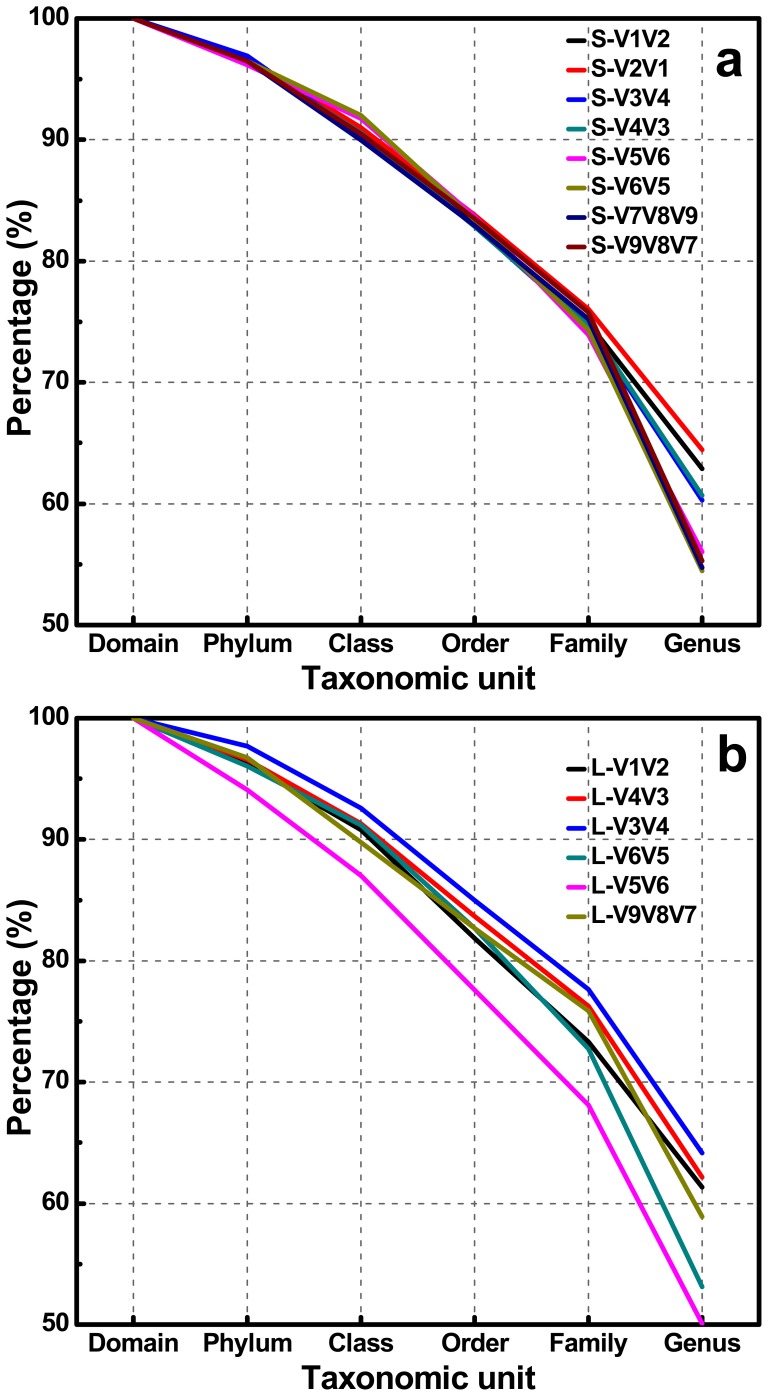
S-type and L-type pyrotags classified into six different taxonomic units assigned by RDP Classifier at 50% bootstrap cutoffs. The equal sequencing depths of 23609 reads and 3276 reads were subsampled to make fair comparison for S-type and L-type pyrotags, respectively.

**Figure 4:**
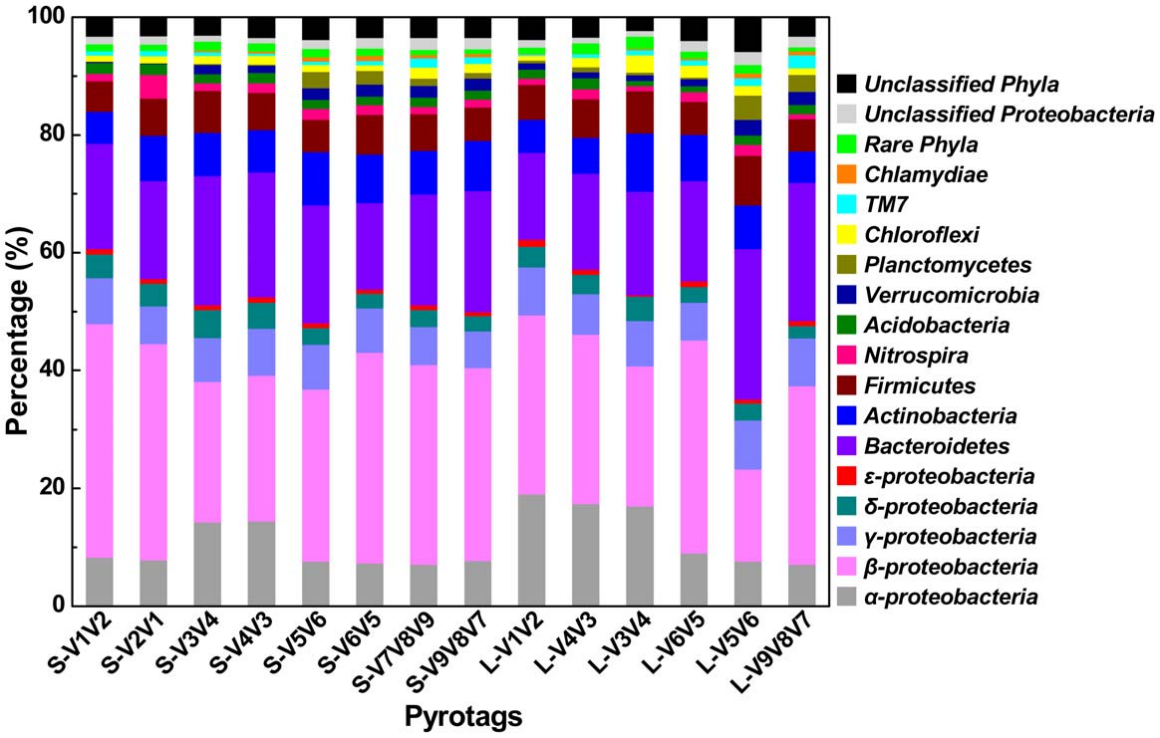
Relative abundance at phylum or class (only for *Proteobacteria*) level assigned by RDP Classifier at 50% confidence thresholds. The extremely low percentage phyla of ‘*Aquificae*, *BRC1*, *Caldiserica*, *Chlorobi*, *Cyanobacteria*, *Deferribacteres*, *Deinococcus-Thermus*, *Fibrobacteres*, *Fusobacteria*, *Gemmatimonadetes*, *Lentisphaerae*, *OD1*, *OP10*, *OP11*, *Spirochaetes*, *SR1*, *Synergistetes*, *Tenericutes*, *Thermotogae*, and *WS3*’ were not displayed in detail and summarized as rare phyla.

**Figure 5:**
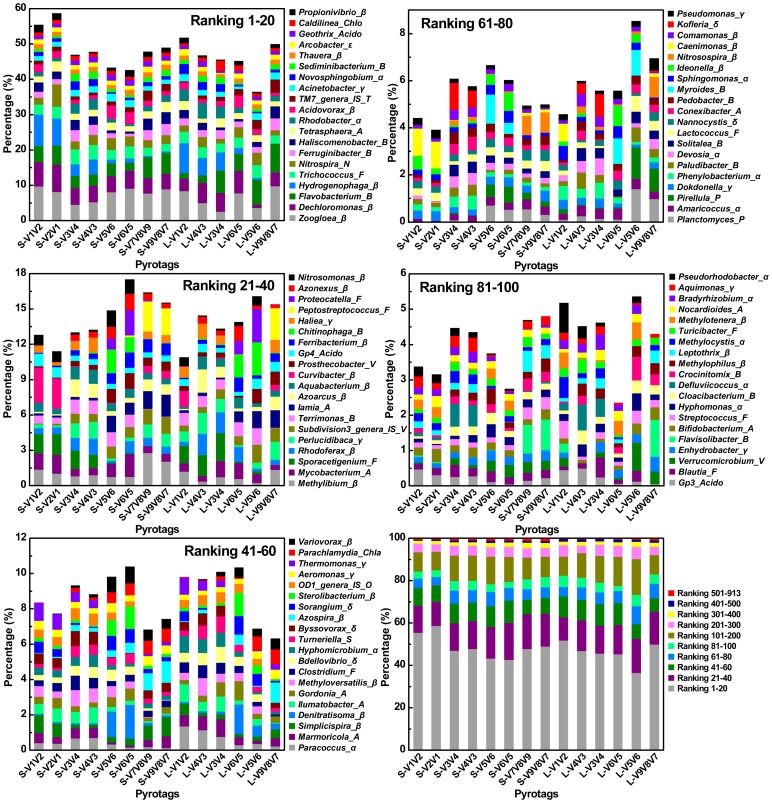
Relative abundance of Top 100 genera assigned by RDP Classifier at 50% bootstrap cutoffs and percentage of ten divided subsets for all ranked genera. The genera were ranked based on the average relative abundance for each pyrotag. All rankings in each subfigure were sorted from high to low level and displayed in the right column from bottom to top accordingly. For instance, Ranking 1, 2, 3, …, and 100 represented genera of *Zoogloea*, *Dechloromonas*, *Flavobacteria*, …, and *Pseudorhodobacter*, respectively. Greek letters of *α*, *β*, *γ*, *δ*, or *ε* modified in the terminus of genus name represented classes of *α-*, *β-*, *γ-*, *δ-*, or *ε- Proteobacteria*, respectively. Similarly, *A*, *Acido*, *B*, *Chla*, *Chlo*, *F*, *N*, *O*, *P*, *S*, *T*, and *V*, indicated phyla of *Actinobacteria*, *Acidobacteria*, *Bacteroidetes*, *Chlamydiae*, *Chloroflexi*, *Firmicutes*, *Nitrospira*, *OD1*, *Planctomycetes*, *Spirochaetes*, *TM7*, and *Verrucomicrobia*, respectively. The total number of these Top 100 genera assigned into the phyla/class was summarized as: *α* (13), *β* (27), *γ* (9), *δ* (5), *ε* (1), *A* (9), *Acido* (3), *B* (13), *Chla* (1), *Chlo* (1), *F* (9), *N* (1), *O* (1), *P* (2), *S* (1), *T* (1), and *V* (3).

OTU-based analysis of rarefaction curves with different cutoffs revealed a high level of species richness in activated sludge (Figure 2). Assigned by RDP Classifier at a 50% cutoff threshold, pyrotags classified into six different taxonomic units indicated high levels of unclassified or unknown bacteria contained in the sample (Figure 3 and Figure S1). Similarly, classification-based analysis also displayed a large bacterial diversity including 31 phyla (Figure 4) and 913 genera (Figure 5) for equal depth subsampled pyrotags, as well as 31 phyla (Figure S2) and 1010 genera (Figure S3) for all clean pyrotags. According to nomenclatural taxonomy and Bergey’s Manual, the current RDP Classifier data set has 39 phyla and 1712 genera, most of which are covered by this study. All these consistent results demonstrated that bacteria populated in activated sludge with a large diversity.

### Diversity Metrics Highly Depends on Primer Sets and Covered Regions

**Figure 6:**
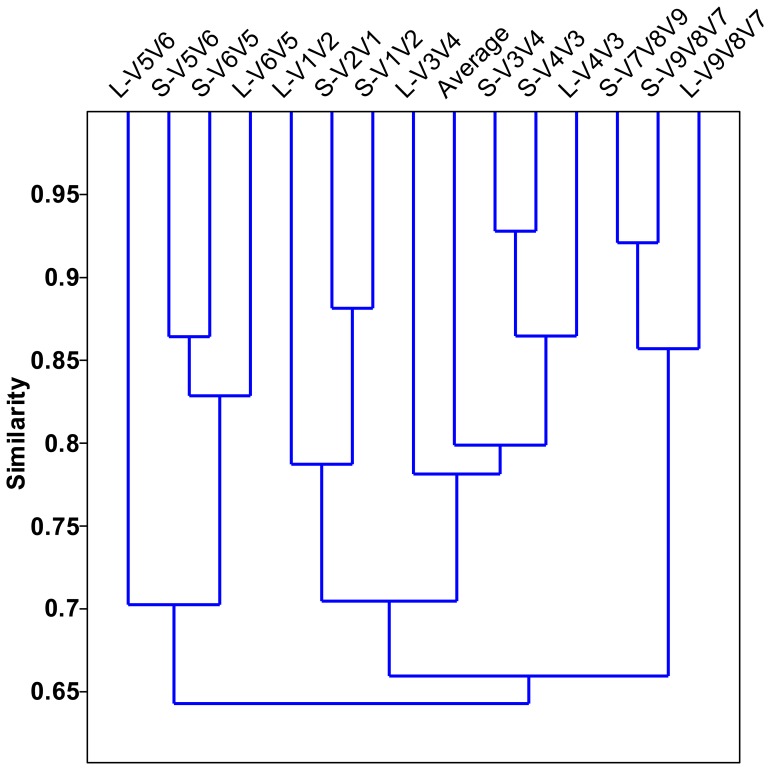
Cluster analysis of all pyrotags based on relative abundance of Top 100 genera. ‘Average’ represented the average relative abundance. The Past statistical software was used to calculate the distance using Bray-Curtis similarity measure.

In this study, 7 primer sets paired by 1 of 4 forward primers (V1F, V3F, V5F, and V7F) and 1 of 4 reverse primers (V2R, V4R, V6R, and V9R) were used to create 7 amplicons and 14 pyrotags (Figure 1 and Table 1). According to the used primer sets and available 454 read length, the covered 16S rRNA gene hypervariable regions were divided into four groups: V1V2 (S-V1V2, S-V2V1, L-V1V2), V3V4 (S-V3V4, S-V4V3, L-V4V3, L-V3V4), V5V6 (S-V5V6, S-V6V5, L-V6V5, L-V5V6), and V7V8V9 (S-V7V8V9, S-V9V8V7, L-V9V8V7). Rarefaction curves of 97%, 94%, and 90% cutoffs (Figure 2) showed minor biased OTU numbers among pyrotags covering regions of V3V4, V5V6, and V7V8V9, but significantly decreased OTU numbers of pyrotags containing V1V2 region. At the phylum/class level (Figure 4), *α-*, *β-Proteobacteria*, and *Bacteroidetes* varied more, while *γ-*, *δ-*, *ε-Proteobacteria*, *Actinobacteria*, and *Firmicutes* varied less. Detection of *Verrucomicrobia*, *Planctomycetes*, and *Chlamydiae* was highly dependent on the choice of primer sets and the covered regions while detection of *Nitrospira*, *Acidobacteria*, *Chloroflexi*, and *TM7* was not. The taxonomic results at the genus level (Figure 5) revealed more diversity dependence on primer sets and covered regions. Among the Ranking 1–20 genera, the abundance in most of them were highly varied with the 16S regions, such as *Hydrogenophaga* (V1V2> V3V4> V5V6> V7V8V9), *Ferruginibacter* (V3V4 or V7V8V9> V1V2 or V5V6), *Haliscomenobacter* (V7V8V9> V1V2 or V3V4> V5V6), and *Acidovorax* (V5V6> V1V2> V3V4 or V7V8V9). A large number of genera assigned in Ranking 21–100 also showed the varied abundance was closely dependent on the selected 16S regions. For example, *Sporacetigenium* (Ranking 23) showed high abundance for V1V2 and V3V4, extremely low abundance for V5V6, and no detection for V7V8V9. However, *Peptostreptococcus* (Ranking 37) displayed high abundance for V7V8V9 but no detection for V1V2, V3V4, and V5V6. Similar cases also occurred for *Terrimonas*, *Iamia*, *Azoarcus*, *Curvibacter*, *Chitinophaga*, *Haliea*, and *Proteocatella* in Ranking 21–40 and other genera not mentioned here in Ranking 41–100. Such inconsistencies could be largely attributed to the used primer sets and covered regions. The similarity of all pyrotags was clustered based on the relative abundance of Top 100 genera in Figure 6 which could reflect the overall variation. The pyrotags covering the same region (V1V2, V3V4, V5V6, or V7V8V9) were clustered together with the first priority, of which the paired pyrotags derived from each short amplicon shared the closest distance.

### Paired Pyrotags Derived from Each Short Amplicon Reveal Biased Diversity Metrics

**Figure 7:**
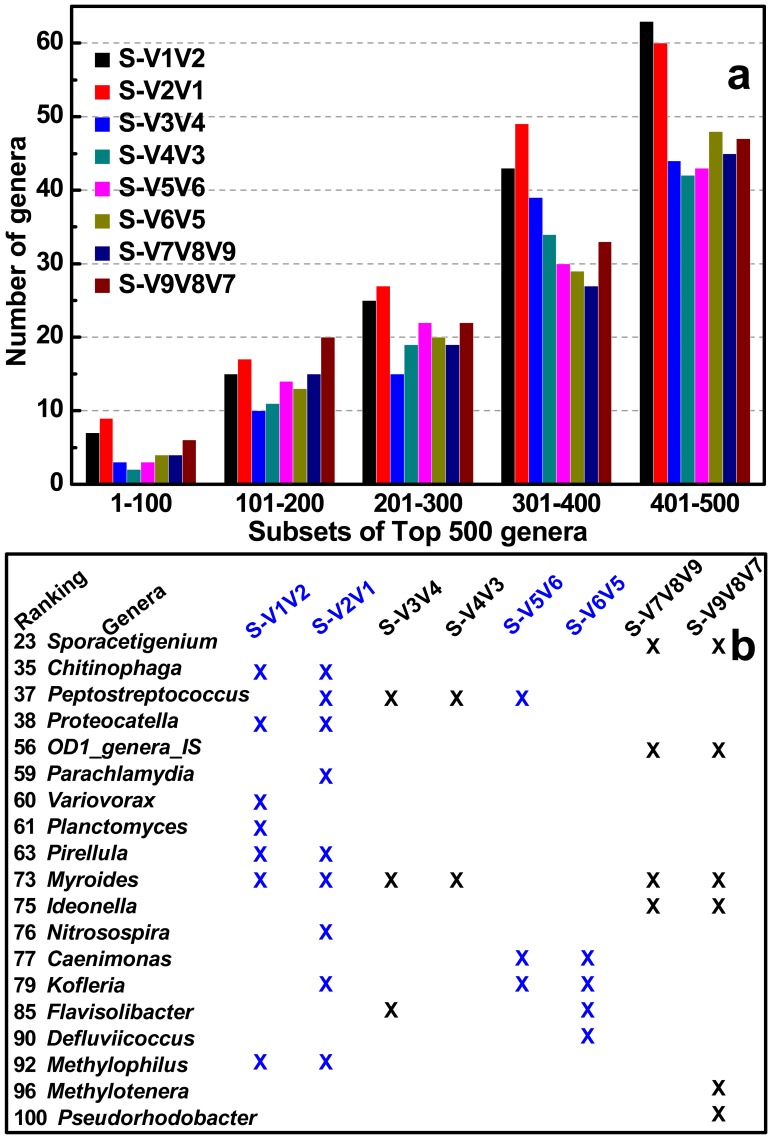
Statistical data of Top 500 (a) and Top 100 (b) genera not detected for each S-type pyrotag. Every subset of 100 genera was divided and calculated independently (a). Genera detected by all S-type pyrotags simultaneously were not listed and the not detected genera were all marked with crosses (b).

In this study, four short amplicons (Amplicon 1, 3, 5, and 7, as described in Figure 1) that were randomly modified with 454 adaptors after PCR to achieve equal chance to sequence-through from either forward or reverse terminus showed biased diversity with varying degrees. This phenomenon was unexpected since no biases were introduced in the steps of PCR and ligation. Each paired pyrotags (e.g. S-V1V2 and S-V2V1) had no PCR bias at all since they were sequenced using the same PCR product (e.g. Amplicon 1). To avoid the ligation bias, the terminuses of all amplicons were modified with nucleotide ‘G’ by fusing it into the 5′ terminus of each primer (Table 1). At the phylum/class level (Figure 4), pairs of S-V3V4 & S-V4V3 and S-V7V8V9 & S-V9V8V7 that were derived from Amplicon 3 and Amplicon 7 respectively, showed minor differences when comparing any one pair independently. However, pairs of S-V1V2 & S-V2V1 (Amplicon 1) and S-V5V6 & S-V6V5 (Amplicon 5) displayed major biases during each pair internal comparison. For instance, S-V2V1 surveyed the abundance of *Nitrospira* several-fold higher than that investigated by S-V1V2. Comparison of each paired pyrotags derived from the four short amplicons indicated that *α-*, *γ-*, *δ-*, and *ε-Proteobacteria* matched well, and less biases occurred for *Actinobacteria* and *Firmicutes*, but more biases occurred for *β-Proteobacteria* and *Bacteroidetes*. At the genus level (Figure 5), such biases between each group of paired pyrotags were displayed even more obviously. Moreover, the biased extent and depth seemed to be dependent on genus specificity and covered regions. Four genera of *Nitrospira* (Ranking 6), *Tetrasphaera* (Ranking 9), *Haliea* (Ranking 36), and *Nocardioides* (Ranking 97) showed major differences between paired pyrotags of S-V1V2 & S-V2V1. Six genera of *Propionivibrio* (Ranking 20), *Rhodoferax* (Ranking 24), *Proteocatella* (Ranking 38), *Myroides* (Ranking 73), *Ideonella* (Ranking 75), and *Methylophilus* (Ranking 92) significantly differed in paired pyrotags of S-V5V6 & S-V6V5, of which *Ideonella* and *Methylophilus* had additional major biases in S-V1V2 & S-V2V1 and S-V7V8V9 & S-V9V8V7, respectively. Figure 7B further illustrated the detection biases of the Top 100 genera. However, it is noteworthy to point out that most genera exhibited favorable matches or less differences within each paired pyrotags. The comparison at both phylum/class and genus levels was concluded that V1V2 and V5V6 regions appeared to be inclined to produce biases rather than V3V4 and V7V8V9 regions, also demonstrated by the cluster analysis in Figure 6.

### Determination of Abundant Bacteria Using Relative Abundance does not Rely on the Sequencing Depth

From the same sequencing depths (subsampled 23,609 reads for S-type pyrotags and 3276 reads for L-type pyrotags) to original sequencing depths (including all clean reads after filtration), the number of each pyrotag used for downstream analysis increased 53.6% (S-V1V2), 58.0% (S-V2V1), 37.0% (S-V3V4), 60.3% (S-V4V3), 233.8% (S-V5V6), 193.7% (S-V6V5), 82.1% (S-V7V8V9), 0% (S-V9V8V7), 41.8% (L-V1V2), 36.4% (L-V4V3), 0% (L-V3V4), 123.2% (L-V6V5), 163.2% (L-V5V6), and 171.7% (L-V9V8V7), respectively (Table 2). Although the percentages of analyzed reads increased at significantly different levels (from 0 to 233.8%), all profiles of corresponding pyrotags were compared to reveal very slight differences at both the phylum/class level (Figure 4 *vs* Figure S2) and Top 100 genus level (Figure 5 *vs* Figure S3). In addition, the classification curves (Figure 3 *vs* Figure S1) also displayed similar profiles at six different taxonomic units. It is implied that high sequencing depth made few contributions to the determination of abundant bacteria. The total of 208,528 reads which subsampled for equal depth analysis covered 913 genera, while all trimmed clean reads which contained 395,718 reads covered 1010 genera. The reads nearly doubled, but only 97 genera increased. Moreover, none of 97 genera were belonged to the Top 500 ranking genera which accounted for more than 99% of the classified reads (Figure 5). In conclusion, relative abundance analysis indicated that the sequencing depth affected the determination of rare bacteria but not abundant bacteria.

### Primers of V1F and V2R Underestimate Diversity Metrics

Comparing all pyrotags in this study, three of them (including S-V1V2, S-V2V1, and L-V1V2) seemed to underestimate the diversity of composite activated sludge sample. They were all covered by V1V2 region, which were derived from two primer sets of V1F & V2R and V1F & V4R. Rarefaction curves at three different cutoffs all revealed similar trends for most pyrotags except for S-V1V2, S-V2V1, and L-V1V2, which appeared to significantly underestimate the diversity (Figure 2). At the phylum level (Figure 4), S-V1V2 and S-V2V1 displayed the incapacities of detecting the phyla of *Verrucomicrobia*, *Planctomycetes*, and *Chlamydiae*. Among the Top 500 genera, S-V1V2 and S-V2V1 remarkably showed more genera not detected than other pyrotags in every 100 divided ranking subset (Figure 7A). Both S-V1V2 and S-V2V1 occupied the highest percentages (over 80%, the statistical subfigure of Figure 5) of the Top 100 genera, but the least genera covered (Figure 7B). Figure 3A showed that the highest percentages of reads could be classified at genus level for S-V1V2 and S-V2V1, implying that the most classified reads covered the least genera. In conclusion, both OTU-based and classification-based analyses demonstrated the worst performance of V1F and V2R that could result in the underestimate of diversity metrics.

### Primer Set of V3F and V4R is Highly Recommended for Future Studies

Summarizing the results, V3V4 region covered by the primer set of V3F and V4R showed the best performance: i) less biases occurred between S-V3V4 and S-V4V3 at both phylum/class and genus levels (Figure 4 and Figure 5); ii) V3F and V4R covered more genera than other primer sets (Figure 7); and iii) the profile of average abundance of Top 100 genera for the 14 pyrotags were clustered with V3V4 region (Figure 6). To achieve relatively accurate diversity metrics, here we highly recommended V3F and V4R as the preferred primer set in future studies.

## Discussion

Although conventional isolation and collection of 16S rRNA genes has been continuously enlarging the 16S rRNA gene database and our understanding on microbial diversity, it is not sufficient to profile the microbial communities in highly complex environments. To solve this problem, pyrosequencing of PCR amplified 16S rRNA gene fragments has been demonstrated as the current most promising approach [24], [25]. However, more attention should be paid to the selected 16S primer sets since different primer sets can give biased diversity pictures, as illustrated by the results of this and other studies [12]–[14], [26]–[28]. Although activated sludge has been displayed to be populated by highly diverse bacteria and serves as a representative sample in this methodology study, we believe that another environmental sample with a high bacterial diversity may also be equally suitable to this study. The abundant bacteria at both phylum and genus levels revealed in this study showed similar profiles with several case studies on activated sludge using 16S V4 pyrosequencing [16], [29]. The experimental design of this study mainly focuses on the current 454 FLX Titanium version (∼400 bp, four short amplicons) and the future 454 FLX+ upgraded version (∼700 bp, three long amplicons). Potential deviations may exist within the pyrotags derived from long amplicons, since less clean reads were obtained for downstream analysis, especially for subdominant bacteria since the limited reads covered them randomly. However, the profile of abundant bacteria was less affected by the sequencing depth, as demonstrated in this study.

Generally, the sequencing direction should not produce biased diversity determination, but in this study this phenomenon occurred in 4 groups of paired pyrotags obtained from 4 short amplicons, especially for S-V1V2 & S-V2V1 and S-V5V6 & S-V6V5. It is not due to the PCR bias since the 454 adaptors were modified to the same PCR product for each paired pyrotags. Theoretically, it is possible to introduce bias at the ligation step, but such probability is very low as pointed out by Claesson et al [12]. Moreover, the 5′ terminus of each amplicon was modified with nucleotide ‘G’ (Table 1) and the 3′ terminus was introduced with nucleotide ‘A’ during PCR, so the same terminal frame (5′-CA-3′) was formed for TA ligation using Roche 454 kit (like TA cloning model) with equal possibility. The most likely explanation is largely attributed to the innate sequencing bias of Roche 454 platform, for instance, sequencing from one terminus works well but sequencing from the reverse terminus might be interrupted. Here, it is of particular importance to note that 454 platform only works on two kinds of fragments: A-adaptor fused to 5′ terminus & B-adaptor fused to 3′ terminus (reading from forward primer end) and A-adaptor fused to 3′ terminus & B-adaptor fused to 5′ terminus (reading from reverse primer end), but it does not work on those fragments with the same adaptor (A or B) fused to both 5′ and 3′ terminuses. It is necessary to carry out further investigation to clarify this interesting phenomenon.

In most cases, it is well accepted that a longer 16S fragment carries more classification information and less discrimination in the phylogenetic analysis. However, it is not always true and depends on the frequency of nucleotide substitution during the long-term evolution. As demonstrated in this study, although the average length of V5V6 region (242 bp) is much shorter than V3V4 region (415 bp), it has relatively equal ability to assign reads into genus level, at least for the Top 100 genera (Figure 7B). For instance, both regions of V3V4 (*Peptostreptococcus* and *Myroides*) and V5V6 (*Caenimonas* and *Kofleria*) have two genera out of detection. The V5V6 region carrying sufficient classification resolution on a shorter length is very promising and could be applied in the improved Illumina paired-end sequencing platform [30] by assembling paired-end reads to obtain the complete length of V5V6 region.

It is not surprising that S-V1V2 and S-V2V1 underestimated the diversity metrics. Several possible explanations might be useful in understanding this problem. Firstly, as mentioned above, V1V2 region may not carry enough classification resolution to make accurate assignment, though it is longer than V5V6 region. Secondly, the primer set of V1F and V2R covers limited bacterial populations experimentally. In this study, an abnormal phenomenon was observed that the Top 4 genera including *Zoogloea*, *Dechloromonas*, *Flavobacteria*, and *Hydrogenophaga* displayed a total of ∼30% abundance in both S-V1V2 and S-V2V1, while other pyrotags only revealed less than 20% (Figure 5). Therefore, the over-amplified proportions of some bacterial populations leading to the repression of other bacterial populations might be another interpretation. However, these potentials need to be validated by further investigation.

Roche 454 pyrosequencing technology usually has advantages over other sequencing platforms applied in microbial ecological studies to explore global diversities and monitor population shifts [6]. Accurate determination of microbial diversity using 16S pyrotags could be influenced by a number of factors, such as sample preparation, DNA isolation, primer set selection, PCR amplification, etc. 16S primer set selection is just one determinant, but perhaps the most important one. Many efforts have been paid to figure out which primer set or region could offer the most accurate taxonomic assignment. Different groups reported different results supported by experiments or/and *in silico* predictions that is largely influenced by the samples [9]–[14]. For example, Claesson et al. reported that V3V4 and V4V5 regions were supported by *in silico* evaluation but experimental pyrosequencing of V3V4 showed the worst performance compared to other 16S regions [12]. It is due to the fact that they used human feces as the studied sample which revealed a low bacterial diversity that was completely different from activated sludge. According to our findings, here we highly recommend the combined V3 and V4 regions using primer set of V3F and V4R, though only a few studies employed this primer set [12], [31] while primer sets targeting single V3 or V4 region are used more frequently [32]–[35]. In addition, V1F and V2R are not recommended, even though they were widely used in early studies [36], [37]. In conclusion, it is impossible to achieve unbiased diversity metrics, but it is possible to set up standard approaches to reach this goal and compare microbial diversity from different environments fairly.

## Supporting Information

Figure S1 **The same figure legend with** **Figure 3** **.** All trimmed clean reads were used for analysis.
(TIF)

Figure S2 **The same figure legend with** **Figure 4** **.** All trimmed clean reads were used for analysis.
(TIF)

Figure S3 **The same figure legend with** **Figure 5** **.** All trimmed clean reads were used for analysis.
(TIF)
