# Supplementary figures and images for: Biased Diversity Metrics Revealed by Bacterial 16S Pyrotags Derived from Different Primer Sets
Source: PLoS One. 2013 Jan 14;8(1):e53649. doi: 10.1371/journal.pone.0053649 (PMC3544912; doi:10.1371/journal.pone.0053649)

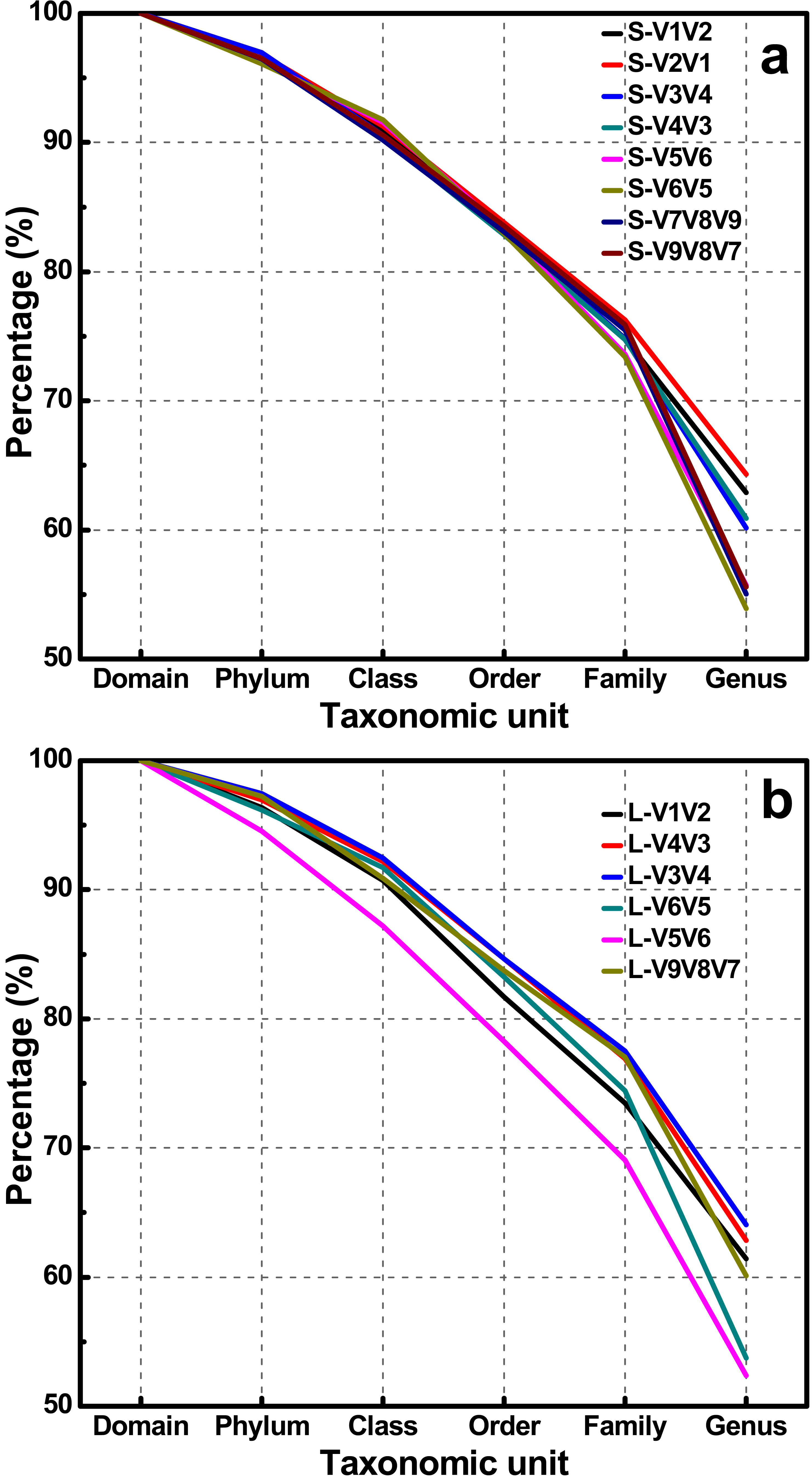

Supplement: Figure S1 — The same figure legend with Figure 3 . All trimmed clean reads were used for analysis. (TIF) [file pone.0053649.s001.tif]

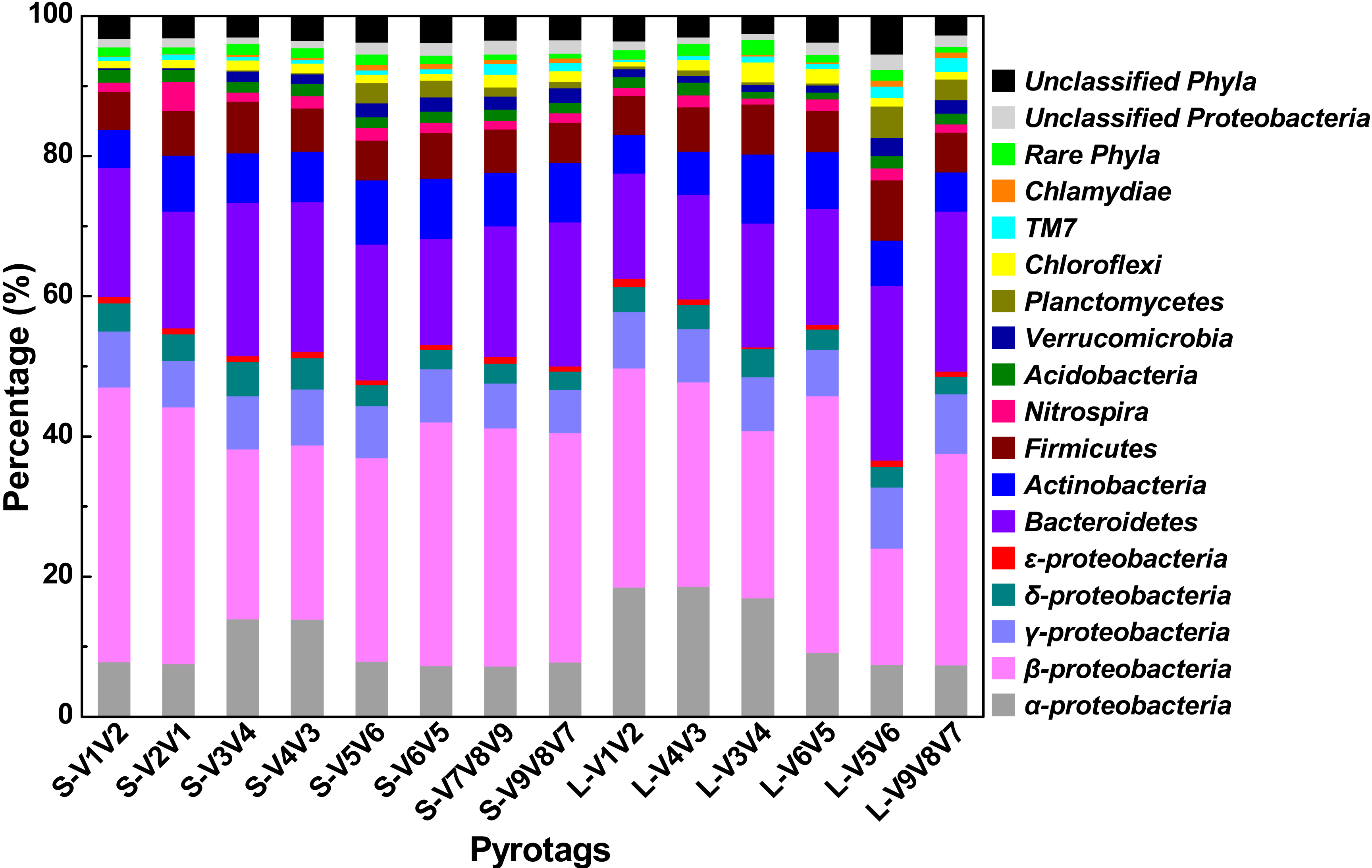

Supplement: Figure S2 — The same figure legend with Figure 4 . All trimmed clean reads were used for analysis. (TIF) [file pone.0053649.s002.tif]

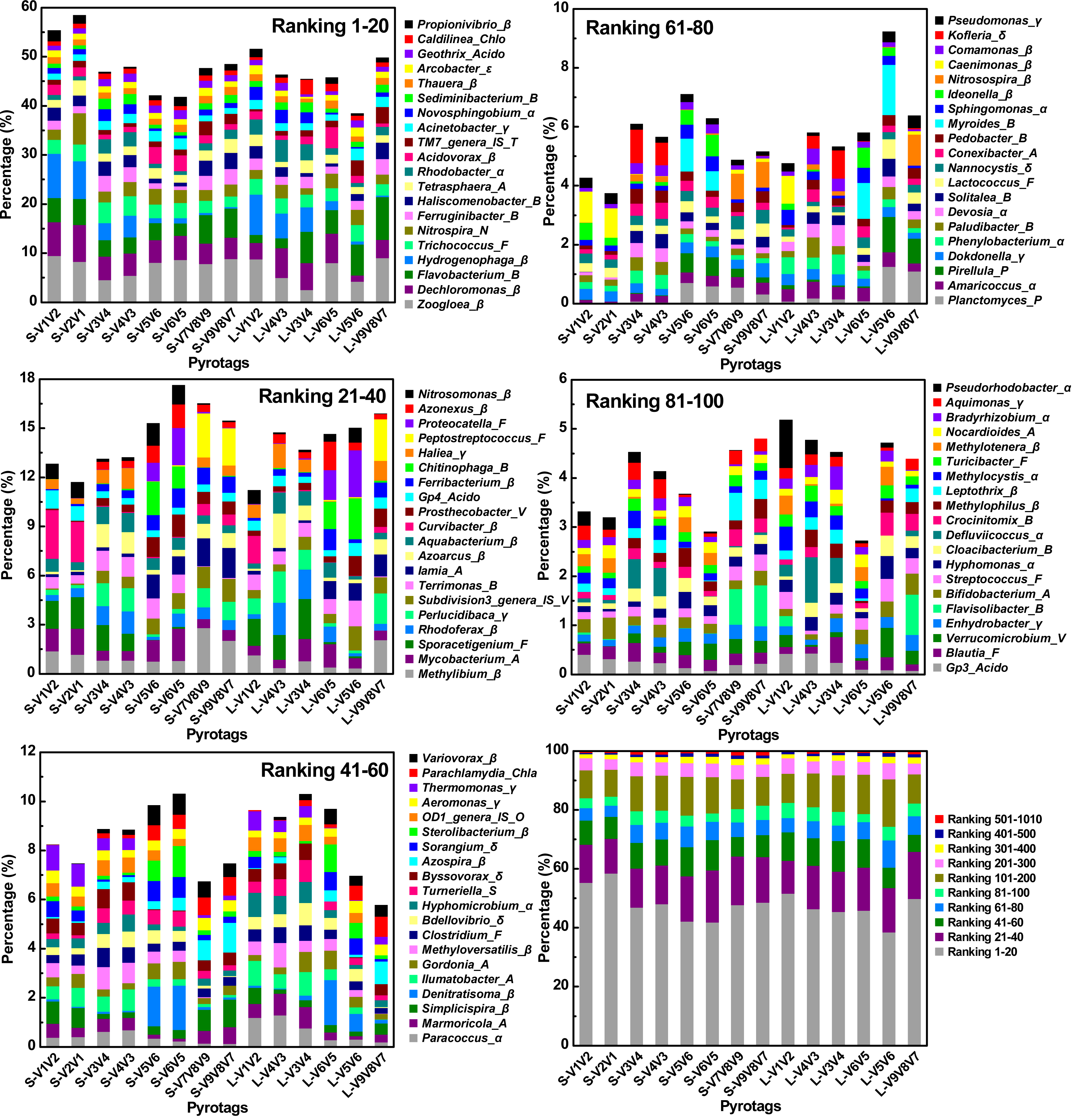

Supplement: Figure S3 — The same figure legend with Figure 5 . All trimmed clean reads were used for analysis. (TIF) [file pone.0053649.s003.tif]
